# The cardiovascular determinants of physical function in patients with end-stage kidney disease on haemodialysis

**DOI:** 10.1007/s10554-020-02112-z

**Published:** 2020-11-30

**Authors:** Sherna F. Adenwalla, Roseanne E. Billany, Daniel S. March, Gaurav S. Gulsin, Hannah M. L. Young, Patrick Highton, Darren C. Churchward, Robin Young, Alysha Careless, Clare L. Tomlinson, Gerry P. McCann, James O. Burton, Matthew P. M. Graham-Brown

**Affiliations:** 1 Department of Cardiovascular Sciences, University of Leicester and NIHR Leicester Cardiovascular Biomedical Research Centre, Glenfield Hospital, Leicester, UK; 2 John Walls Renal Unit, University Hospitals Leicester NHS Trust, Leicester, UK; 3 NIHR Leicester Biomedical Research Centre, University Hospitals of Leicester NHS Trust, Leicester, UK; 4 Department of Respiratory Sciences, University of Leicester, Leicester, UK; 5 Research and Innovation Department, University Hospitals Leicester NHS Trust, Leicester, UK; 6 Robertson Centre for Biostatistics, University of Glasgow, Glasgow, UK; 7 National Centre for Sport and Exercise Medicine, School of Sport, Exercise and Health Sciences, Loughborough University, Loughborough, UK

**Keywords:** Physical activity, MRI, ESKD, Global native T1, Cardiovascular function

## Abstract

Patients with end-stage kidney disease (ESKD) are often sedentary and decreased functional capacity associates with mortality. The relationship between cardiovascular disease (CVD) and physical function has not been fully explored. Understanding the relationships between prognostically relevant measures of CVD and physical function may offer insight into how exercise interventions might target specific elements of CVD. 130 patients on haemodialysis (mean age 57 ± 15 years, 73% male, dialysis vintage 1.3 years (0.5, 3.4), recruited to the CYCLE-HD trial (ISRCTN11299707), underwent cardiovascular phenotyping with cardiac MRI (left ventricular (LV) structure and function, pulse wave velocity (PWV) and native T1 mapping) and cardiac biomarker assessment. Participants completed the incremental shuttle walk test (ISWT) and sit-to-stand 60 (STS60) as field-tests of physical function. Linear regression models identified CV determinants of physical function measures, adjusted for age, gender, BMI, diabetes, ethnicity and systolic blood pressure. Troponin I, PWV and global native T1 were univariate determinants of ISWT and STS60 performance. NT pro-BNP was a univariate determinant of ISWT performance. In multivariate models, NT pro-BNP and global native T1 were independent determinants of ISWT and STS60 performance. LV ejection fraction was an independent determinant of ISWT distance. However, age and diabetes had the strongest relationships with physical function. In conclusion, NT pro-BNP, global native T1 and LV ejection fraction were independent CV determinants of physical function. However, age and diabetes had the greatest independent influence. Targeting diabetic care may ameliorate deconditioning in these patients and a multimorbidity approach should be considered when developing exercise interventions.

**Electronic Supplementary Material:**

The online version of this article ( 10.1007/s10554-020-02112-z ) contains supplementary material, which is available to authorized users.

## Introduction

Chronic kidney disease (CKD) is associated with progressive decline in exercise capacity and ability to perform activities of daily living, with patients on haemodialysis amongst the most sedentary of all patient groups [1]. The reasons for this functional decline are complex but related to: reduced cardiorespiratory fitness, declining levels of self-efficacy, muscle wasting and an increasing symptom burden, leading to a deconditioning spiral which exacerbates sedentary behaviour [1]. The enforced sedentary time during haemodialysis itself is also important to note. Studies suggest that not only do objective measures of physical function predict mortality and cardiovascular (CV) events for this patient group, but there is now evidence to suggest that interventions which improve physical performance also improve clinical outcomes, including mortality [2, 3].

It is well documented that patients with end-stage kidney disease (ESKD) have significantly elevated CV risk [4]. This excess CV risk is related to pathological, stereotyped changes in CV structure and function, including left ventricular hypertrophy (LVH), left ventricle (LV) dilatation, myocardial fibrosis and aortic stiffness. Collectively termed uraemic cardiomyopathy (UC) [5], these maladaptations link closely to mortality [6, 7]. Multiparametric cardiovascular magnetic resonance imaging (CMR) can comprehensively phenotype prognostically relevant pathological aspects of UC with excellent reproducibility [6, 8, 9].

The relationships between prognostically significant measures of CV disease (CVD) and physical function in patients with ESKD have not been fully explored. Establishing the CV determinants of physical functioning would offer insight into how exercise interventions may improve CV outcomes for these patients. This could guide development of studies seeking to mitigate CV risk through lifestyle interventions.

This study investigated the relationship between prognostically relevant measures of CV structure and function, assessed with CMR and cardiac biomarkers, and objective measures of physical function in patients on haemodialysis. These data will offer valuable insight into the relationships between core outcome measures for haemodialysis patients, as identified by the SONG-HD initiative [10].

## Methods

The data from this study is taken from the baseline CMR scans and associated clinical, biochemical and physical functioning data of the 130 patients on haemodialysis recruited to the CYCLE-HD trial (ISRCTN11299707). This was a randomised controlled trial investigating the impact of six months of intradialytic cycling on CV structure and function, assessed by CMR, in patients on maintenance haemodialysis. Inclusion and exclusion criteria were as previously described [11]. Prevalent haemodialysis patients over the age of 18 were included whilst those unfit to undertake exercise according to the American College of Sports Medicine Guidelines or undergo MRI scanning were excluded. The study was given ethical approval by the NHS Research Ethics Committee East Midlands (Northampton; REC ref: 14/EM/1190). All data included for analysis were collected prospectively, entered into an electronic case report form and were analysed at the Robertson Centre for Biostatistics, University of Glasgow, United Kingdom. The trial sponsor was the University of Leicester.

### Physical function assessments

The incremental shuttle walk test (ISWT) is highly correlated with V̇O_2_ peak, which is the gold-standard measure of aerobic capacity and associated with mortality in the haemodialysis population [12]. The ISWT also has excellent test-retest reliability in CKD populations [13]. The sit-to-stand 60 (STS60) assesses lower-extremity strength and endurance. Declining estimated glomerular filtration rate (eGFR) correlates well with declining performance in this physical function test [14], and has proven reliability in patients with CKD [13].

Field-tests of physical function were completed on non-dialysis days and not after the long-break. Trained research staff performed all assessments following standard operating procedures. For the ISWT, participants walked between two cones placed nine metres apart and walking speed was guided by a recorded bleep sound [13]. The STS60 was assessed by asking participants to stand up from a chair as many times as possible in 60 s [13]. Familiarisation tests were performed prior to the main protocol to account for any learning effect. Adequate break was given between each test.

### Cardiovascular phenotyping

All subjects underwent comprehensive cardiovascular phenotyping with a CMR scan and blood tests for circulating markers of cardiovascular disease. Left ventricular mass index (LVMi); LV ejection fraction [15]; LV mass:volume ratio; global native T1 (measure of myocardial fibrosis); pulse wave velocity (PWV); and global longitudinal strain (GLS) were acquired with CMR and high-sensitivity troponin I (hsTnI) and N-Terminal Pro-BNP (NT-proBNP) were assessed from plasma.

#### CMR imaging protocol

**Fig. 1:**
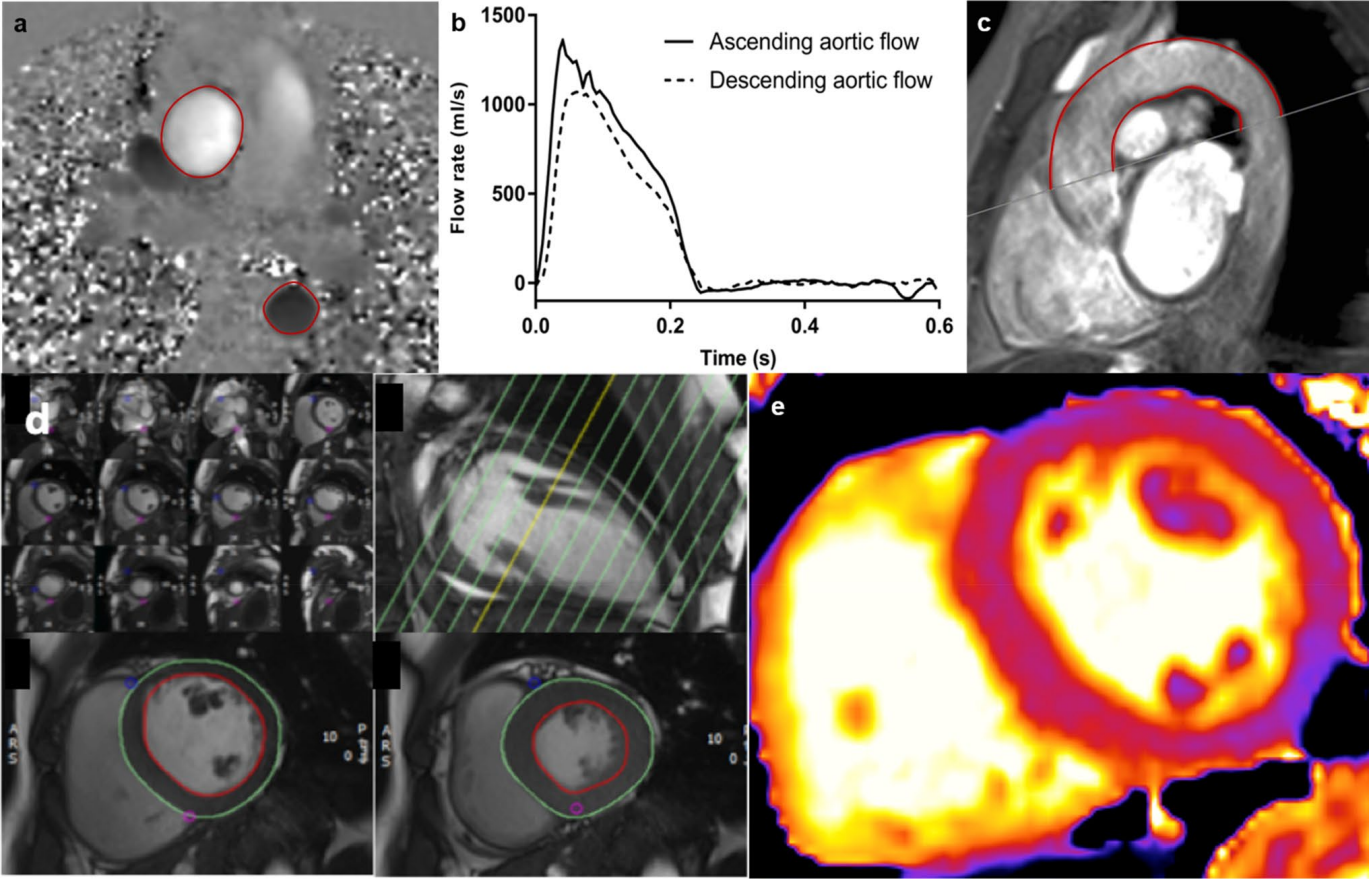
Assessment of pulse wave velocity using two-dimensional phase*-*contrast CMR. For the PWV calculation, axial aortic contours were mapped onto phase–contrast cines (**a**), allowing the waveform transit time to be calculated from flow curves of the ascending and descending aorta (**b**). Distance was measured using a sagittal–oblique cine. Outer and inner borders of the aortic arch were manually drawn and the mean distance of these two borders was calculated (mm) (**c**). LV mass and volumes measured from a contiguous short-axis stack of cine images planned from long-axis view with endo and epicardial contours drawn at end-diastole and end-systole (**d**). Native T1 mapping of a short-axis ventricular slice of the left ventricle for assessment of myocardial fibrosis (**e**)

Complete LV and aortic functional analysis was undertaken on a 3T CMR platform (Skyra, Siemens Medical Imaging, Erlangen, Germany) with an 18-channel phased-array receiver coil. To standardise participants’ volume status at time of assessment, scans were conducted on a non-dialysis day, but not after the long-break. The CMR protocols for acquiring LV cine imaging, native T1 maps, and PWV conformed to internationally recognized standards [9, 16]. Short-axis cine images covering the LV were taken at 8-mm slice thickness, no gap, field of view 28 × 30 cm, matrix 208 × 256, repetition time 2.9 ms, echo time 1.2 ms, flip angle 64°−79°, temporal resolution < 50 ms, 80% phase, with 30 phases per cardiac cycle, in-plane image resolution 1.1 × 1.5 mm to 1.3 × 1.7 mm [9]. Mid-ventricular native T1 maps of the LV were acquired using the modified look-locker inversion recovery (MOLLI) sequence [16], with 3(3)3(3)5 sampling pattern and the following parameters: slice thickness 8.0 mm, field of view 300 × 400 mm, flip angle 50°, minimum TI 120 ms, inversion-time increment 80 ms. For acquisition of PWV, gradient echo and high temporal resolution, through-plane phase contrast cines were acquired perpendicular to the ascending and descending aorta at the level of the pulmonary artery bifurcation, permitting calculation of transit time for PWV, as previously described [9]. Sagittal-oblique cines of the ascending and descending aorta were captured for distance measurement [17]. Examples of images acquired for analysis are shown in Fig. 1.

#### CMR image analysis

Scans were analysed offline by a single blinded observer (MGB). LV structural and functional analysis, native T1 maps and GLS were analysed using the software package CMR^42^ (Circle Cardiovascular Imaging, Calgary, AB, Canada), as previously described [8, 18]. PWV was analysed using Java Image Manipulation version 6 (Xinapse Systems, Essex, UK) [17].

##### LV volumes, function, parametric mapping and strain analysis

LV volumes and mass were quantified with epicardial and endocardial short axis cines at end-diastole and end-systole. LV mass was indexed to body surface area. For T1 mapping, endocardial and epicardial borders were drawn on basal and mid-ventricular T1 parametric maps. The anterior right ventricular insertion point was defined to automatically divide the basal-ventricular and mid-ventricular slices. After removal of segments affected by artefact, an average T1 time for the whole of the myocardium was calculated from the mean of remaining segments. The average native T1 time for healthy control subjects on this scanner and analysed in this way is 1204 ± 38 ms. Tissue tracking analysis was used to define strain parameters. Global longitudinal strain was assessed by drawing endo- and epicardial contours on LV long-axis cines in end-diastole and defining the LV base and apex.

##### PWV

Every ninth slice of the ascending and descending aorta was manually contoured and propagated using a gradient echo cine. Contours were mapped onto the phase-contrast cine, allowing the temporal shift to be determined. The cut made to define the axial slice for the phase-contrast sequences was superimposed on the sagittal–oblique cine and the average distance measured around the aortic arch. aPWV was calculated as described in Fig. 1.

#### Blood sampling and routine clinical information

Blood samples were collected from the arterial needle before dialysis (but not following the long-break); 9.8 ml of blood was collected into two S-Monovette K_3_ Serum-Gel treated tubes (Sarstedt Monovette, Sarstedt AG & Co, Nümbrecht, Germany) to be centrifugated at 2500 × g for 15 min 20 °C. The serum was pipetted into 2 ml polypropylene micro tubes (Sarstedt Monovette, Sarstedt AG & Co, Nümbrecht, Germany) before being stored at – 80 °C for later analysis. Analysis of hsTnI and NT-proBNP were completed in triplicate as per the manufacturer’s instructions (hsTnI, STAT high sensitive troponin-I, Architect, Abbott Diagnostics, USA; NTPro-BNP, Elecsys proBNP II, Cobas, ROCHE, USA).

Routine clinical information was extracted from medical records. Routine biochemical and haematological data were collected prospectively as well as full past medical history.

### Statistical analysis

Statistical analysis was undertaken using R version 3.4.1 to fit both the univariate and multi-variable linear regression models. Normally distributed data are expressed as mean ± standard deviation and non-normally distributed data are expressed as median (interquartile range), with a log-transformation being applied for subsequent inclusion as an outcome in analysis. Comparisons between subgroups were made using independent t-tests for normally distributed data and Mann U Whitney tests for non-normally distributed data. Pearson correlations were used for correlations of normally distributed data and Spearman correlation coefficients for non-normally distributed data.

Sixteen separate multivariate linear regression models were tested with each physical function test (ISWT and STS60) as the dependent variable and each biomarker of CV health as the independent variable. Models were adjusted for the following pre-determined variables known to influence performance in physical function tests: age [19, 20], body mass index (BMI) [20, 21], gender [20, 21], presence of diabetes mellitus [19], ethnicity and systolic blood pressure (SBP) based on pre-existing literature and biological plausibility. Independent variables were limited in each model to avoid overfitting. For multivariate analysis, data was analysed listwise and no data imputations were performed, hence numbers were lower than the total participants who completed each field-test. In addition to unstandardized beta coefficients, standardized beta coefficients are presented in supplementary data so the impact of variables in each multivariate regression model can be compared.

## Results

Baseline patient demographics, CMR variables, circulating markers of CVD and completeness of datasets are shown in Table 1. Of the 130 participants in the baseline CYCLE-HD cohort, mean age was 57 years (± 15), 73% were male and median dialysis vintage was 1.3 years (0.5, 3.4). A large proportion of patients had a background of hypertension (67%) and diabetes (38%). Subgroup comparisons of field test performance and CV biomarker averages are in Supplementary data, Appendix 1.

**Table 1:** Demographic and baseline data of the CYCLE-HD cohort

	Baseline data of CYCLE-HD cohort n = 130 [N_miss_]
Age (years)	57.2 ± 15
Male, n (%)	95 (73%)
Ethnicity, n (%)	
White	58 (45%)
BAME	72 (55%)
SBP (mmHg)	143 ± 22
DBP (mmHg)	76 ± 14
Dialysis vintage (years)	1.3 (0.5, 3.4)
Haemoglobin (g/L)	111 ± 17
Albumin (g/L)	36.9 ± 5
CRP (mg/L)	14.0 (8, 34)
Total Cholesterol (mmol/L)	4.0 ± 1.4 [28]
Triglycerides (mmol/L)	1.93 ± 1.39 [47]
HbA1c (%)	5.7 (5, 7) [38]
BMI (kg/m^2^)	27 (23, 31)
Co-morbidities	
Ischaemic heart disease, n (%)	16 (12%) [1]
Hypertension, n (%)	86 (67%) [1]
Diabetes mellitus, n (%)	49 (38%) [1]
Atrial Fibrillation, n (%)	5 (4%) [1]
Previous renal transplant, n (%)	20 (15%)
CMR measures of cardiovascular disease	
LVMi (g/m^2^)	60 (50, 76)
LV ejection fraction (%)	53.6 ± 10
LV mass/LV end-diastolic volume (g/mL)	0.73 ± 0.2
Global Native T1 (ms)	1273.6 ± 40.8 [6]
Global longitudinal strain (%)	− 13.2 ± 3.3
PWV (m/s)	8.2 (6, 11) [13]
Humoral markers of cardiovascular disease	
NT pro-BNP (pg/ml)	2693 (1136, 10121) [10]
Troponin I (ng/L)	10.1 (6, 17) [6]
Baseline physical performance in field tests	
ISWT (m)	220 (140, 360) [16]^a^
STS60 (reps)	16.1 ± 11.5 [13]^a^

*[N*_*miss*_*]* number of missing data values. Normally distributed data presented as mean ± SD, non-normally distributed data presented as median (25th, 75th percentile). LV indices indexed to body surface area

*BAME* Black, Asian and minority ethnic, *SBP* systolic blood pressure, *DBP* diastolic blood pressure, *CRP* C-reactive protein, *BMI* body mass index, *LV* left ventricle, *LVMi* left ventricular mass index, *PWV* pulse wave velocity

^a^The missing data for the physical function tests limited the numbers that could be included in univariate and multivariate regression models.

### Univariate associations of CV health with physical performance

Results of the univariate analyses between biomarkers of CV health and the ISWT and STS60 are shown in Table 2. Correlations between NT pro-BNP, LV ejection fraction, LV mass index and global native T1 are demonstrated in Supplementary data, Appendix 2.

**Table 2:** Univariate and multivariate linear regression models to assess cardiovascular determinants of performance in physical function tests

Variable	Outcome	Univariate model			Multivariate model^a^			
		Participants	B (SE)	P Value	Participants	B (SE)	P Value	R^2^
Troponin I (ng/L)^b^	ISWT	111	− 39.84 (13.2)	**< 0.01**	110	− 16.80 (12.0)	0.17	0.38
	STS60	113	− 1.87 (0.9)	**0.03**	112	− 0.23 (0.8)	0.78	0.33
NT pro-BNP (pg/ml) ^b^	ISWT	108	− 22.83 (10.0)	**0.02**	107	− 20.33 (8.9)	**0.02**	0.39
	STS60	110	− 1.24 (0.7)	0.06	109	− 1.34 (0.6)	**0.03**	0.35
LVMi (g/m^2^)	ISWT	114	0.86 (0.8)	0.28	113	− 0.31 (0.7)	0.67	0.39
	STS60	117	0.04 (0.1)	0.46	116	− 0.03 (0.05)	0.52	0.34
LV ejection fraction (%)	ISWT	114	3.18 (1.6)	0.06	113	3.74 (1.4)	**0.01**	0.43
	STS60	117	0.15 (0.1)	0.16	116	0.14 (0.1)	0.15	0.35
PWV (m/s)	ISWT	103	− 9.72 (3.2)	**< 0.01**	102	− 1.10 (3.2)	0.74	0.39
	STS60	106	− 0.47 (0.2)	**0.04**	105	0.11 (0.2)	0.64	0.33
LV mass:volume (g/mL)	ISWT	114	− 50.70 (99.7)	0.61	113	− 46.93 (82.9)	0.57	0.39
	STS60	117	− 1.27 (6.6)	0.85	116	− 0.45 (5.7)	0.94	0.33
Global Native T1 (ms)	ISWT	109	− 1.45 (0.4)	**< 0.01**	108	− 1.29 (0.3)	**< 0.01**	0.48
	STS60	112	− 0.07 (0.03)	**0.01**	111	− 0.06 (0.02)	**0.02**	0.36
Global longitudinal strain (%)	ISWT	114	− 8.20 (5.0)	0.10	116	− 7.97 (4.3)	0.07	0.41
	STS60	117	− 0.48 (0.3)	0.13	112	− 0.27 (0.3)	0.34	0.34

The reference category for gender is ‘male’, for history of diabetes is ‘no’ and for ethnicity is ‘white’

*BMI* body mass index, *LVMi* left ventricular mass index, *LV* left ventricle, *PWV* pulse wave velocity, *ISWT* incremental shuttle walk test, *STS60* sit-to-stand 60

^a^adjusted for age, gender, BMI, diabetic status, ethnicity and systolic blood pressure

^b^Log transformed data. B = unstandardized beta coefficient; SE = standard error of the mean

#### ISWT

On univariate analyses, troponin, NT pro-BNP, PWV and global native T1 were each associated with performance in the ISWT (all p < 0.05). There was some evidence of an association between left ventricular ejection fraction and ISWT, though not to the defined level of statistical significance (p = 0.06).

#### STS60

Troponin, PWV and global native T1 were significant univariate associations with STS60 performance (all p < 0.05). There was some evidence of an association between NT-pro-BNP and the STS60, though not to the defined level of statistical significance (p = 0.06).

### Independent determinants of physical performance

The results of the regressions, adjusted for predetermined variables, between physical function tests and biomarkers of CV health are shown in Table 2. Full regression models showing the impact of age, gender, BMI, diabetes, ethnicity and SBP are presented in Supplementary data, Appendix 3.

#### ISWT

NT pro-BNP, LV ejection fraction, and global native T1 were independent CV determinants of ISWT performance, when adjusted for age, diabetes, gender, BMI, ethnicity and SBP (Table 2). For every 100 ng/L decrease in NT pro-BNP, participants walked 5 m more. A 10% increase in LV ejection fraction translated to a further 3 m walked in the ISWT. For every 10 ms decrease in global native T1, participants were able to walk 8 m more in the field test.

Increasing age, female gender and having diabetes were consistently, significantly associated with worse performance in the ISWT (Appendix 3). Participants with diabetes walked 74–95 m less than those without diabetes.

#### STS60

NT pro-BNP and global native T1 remained independent determinants of STS60 performance after adjustment in the multivariate models (Table 2). For every 100 ng/L decrease in NT pro-BNP, participants were able to do 75 more stands in the STS60. A 10 ms reduction in global native T1 associated with 167 more stands.

Of the adjusting variables, age and diabetes were significantly associated with STS60 performance (Appendix 3). Presence of diabetes was associated with four to six fewer stands in 60 s than those without diabetes.

## Discussion

This is the first study to describe the relationships between prognostically relevant measures of CVD and physical function a in a well-phenotyped cohort of patients with ESKD receiving maintenance haemodialysis. Multiple CV biomarkers were significant univariate determinants of physical function. After adjustment, reduced global native T1, reduced NT pro-BNP and increased LV ejection fraction independently associated with better performance in physical function tests. Age and presence of diabetes had the strongest influence in the multivariate regression models. Consistent with previous literature, female gender was independently associated with worse ISWT performance [1, 21].

### Cardiovascular determinants of the ISWT

Limited exercise tolerance, as measured by the ISWT in this study, is a cardinal manifestation of CVD. On average, our cohort walked 220 m in the ISWT; this is considerably less than reference values for this age group in a healthy population (810 m) [21], but similar to values documented in other patients on haemodialysis [22]. The univariate associations between ISWT and cardiovascular phenotype are all biologically plausible: Poor LV contraction (LV ejection fraction), myocardial ischaemia, fibrosis and aortic stiffness (troponin I, NT pro-BNP, global native T1 and PWV, respectively) would all be expected to associate with blunted aerobic capacity. After adjusting for factors known to limit exercise tolerance, global native T1, NT pro-BNP and LV ejection fraction remained significant determinants of functional capacity.

In our population, higher LV ejection fraction independently associated with distance walked, despite the average LV ejection fraction being only at the lower end of normal. This could be attributed to the spread of values and could explain why the relationship is not particularly influential. If our population included more people with very low ejection fractions, the relationship may have been stronger. There have been no previous studies exploring the association between LV ejection fraction and ISWT performance in this population. The relationship between LV ejection fraction at rest and V̇O_2_ peak have been shown to be insignificant [23], with changes in ejection fraction during exercise playing larger roles [24]. However, these studies were in different populations and used echocardiography to measure ejection fraction.

NT pro-BNP is released in relation to atrial and ventricular dilatation and myocardial wall stress. It is an established prognostic marker in heart failure. Few studies have explored the relationship between BNP and the ISWT, with mixed results [25, 26]. However, NT pro-BNP is a consistent determinant of V̇O_2_ peak across populations of people with cardiovascular disease and can reduce following programmes of exercise [27]. Our findings support the use of NT pro-BNP as an indicator of functional aerobic capacity and suggest it could be a quantifiable marker when targeting exercise interventions in patients with ESKD.

Our study showed a statistically significant independent association between native T1 times, a measure of myocardial fibrosis, and metres walked in the ISWT [16]. This is the first study to examine the relationship between global native T1 values and performance in physical function tests. Ventricular fibrosis, measured by late gadolinium enhancement, has been correlated with reduced V̇O_2_ peak in patients with cardiovascular disease [28]. The only study to this point to look at the relationship between T1 values and V̇O_2_ peak found no significant association [29], although this was in children. The caveat is that histological confirmation that T1 values consistently represent interstitial fibrosis in haemodialysis patients is still lacking. T1 values can also be raised in oedema, amyloid deposition and inflammation [30]. Nevertheless, raised native T1 times indicate significant myocardial tissue abnormality and the independent association with ISWT is an interesting, hypothesis generating finding.

### Cardiovascular determinants of the STS60

A large population-based study found healthy participants aged 55–59 years could do 36 (females) to 41 (males) repetitions of the STS60, a measure of lower extremity strength and endurance [20]. Our cohort’s average was 16 repetitions. This is comparable to data which compared CKD and ESKD patients matched for age, gender, diabetic status, and dialysis vintage [31]. In our study, troponin I, PWV, global native T1 were all univariate associations with the STS60, whilst NT pro-BNP was close to significance. In the adjusted models, NT-pro-BNP and global native T1 were independent determinants of STS60 performance.

There have been no previous studies which have investigated the association between NT-pro-BNP and the STS60 test. An inverse relationship between BNP and muscle mass has been demonstrated in haemodialysis patients [32]. It is possible this reflects sarcopenia and weakness consequent to chronic cardiac stress, however these findings have been upheld even after adjusting for cardiac function [32]. Furthermore, metabolic diseases including obesity and insulin resistance have been associated with decreased production of BNP which could be applicable to our population [33]. The long-term effects of exercise training on natriuretic peptides in ESKD populations needs further study.

We found that native T1 values decreased with increasing numbers of sit-to-stands in our ESKD population. There are no previous studies investigating global native T1 and STS60 or with other measures such as leg strength. However, in line with our findings, T1 values in the myocardium of endurance athletes are lower than in controls [34]. The ability to perform the STS60 is multifactorial. Our participants who performed well at the STS60 had lower T1 values; whether this is due to less myocardial fibrosis is far from certain and any implications of causality needs investigation. Animal models provide early evidence for the role of microRNAs as a causative factor driving myocardial fibrosis and cardiac remodelling through skeletal muscle-heart crosstalk [35], but these results are hypothesis generating.

### Diabetes and physical functioning

Participant age and presence of diabetes consistently had the greatest impact on the multivariate regression models. Those with diabetes performed significantly worse in physical function tests, in line with previous evidence [19]. Cardiovascular disease is the leading cause of mortality in patients with diabetes [36]. This is not solely explained by obesity and a sedentary lifestyle. Patients with T2DM have decreased V̇O_2_ peak compared to similarly obese and sedentary individuals without diabetes [37]. The phenotypic characteristics around exercise impairment in diabetes have been well-explored and include insulin resistance, endothelial dysfunction and impaired mitochondrial function [38]. Unsurprisingly, CV function is affected early in the disease process, with increased native T1 values before the onset of overt ‘diabetic cardiomyopathy’ [39]. Limitations in skeletal muscle due to insulin resistance also factor and lead to functional impairment through sarcopenia. These issues contribute to the overall syndrome of physical deconditioning and accelerated metabolic ageing, and dramatically increase the likelihood of lower physical functioning and frailty [38]. Furthermore, the burden of frailty within the diabetic population is strongly associated with mortality [40].

It is unclear whether the pathophysiological dysfunction leading to poor physical functioning in diabetes is reversible. Interventions such as exercise training and improved glycaemic control can ameliorate subclinical cardiac dysfunction and may be a potential intervention in improving aerobic fitness in this population [41, 42]. The impact of diabetic status on physical functioning in our patients emphasises a pressing need to improve management of diabetes for patients on haemodialysis. By nature of having progressed to ESKD, the patients with diabetes in our study are likely to have profound organ damage elsewhere, such as the heart, brain and muscles, and considerable levels of frailty. It is well established that these patients have a high multimorbidity burden, associated with functional decline and increased mortality. To date, evidence exploring a multimorbidity approach to improve patient outcomes has been limited and interventions have focussed on single conditions. A Cochrane review suggested that designing interventions that target difficulties with functioning may be an effective multimorbidity approach [43]. A logical development would be to factor in multimorbidity when designing and delivering exercise interventions in patients with kidney disease.

### Strengths and limitations

Our study aimed to capture a holistic view of physical performance, by including tests measuring aerobic capacity and lower-extremity strength. In addition, the use of CMR and other biomarkers offers extensive CV phenotyping in this population. Our cohort are representative of a maintenance haemodialysis patient population in the United Kingdom, although females are relatively underrepresented. Field-based physical performance measures are considered a surrogate for ‘gold-standard’ laboratory-based measurements of physiological impairment (such as V̇O_2_ peak). However, field-based tests can give more information about ability to perform activities of daily living and therefore may be more important to the patient. Some studies have found variables such as leg strength and lung function to be associated with physical performance, but such variables were not collected in the original cohort and therefore could not be included.

There was no significant relationship found between GLS and physical function which was somewhat surprising. This may be because of an inherent limitation in the assessment of strain parameters in patients prone to changes in cardiac loading from alterations in systemic volume status. Although efforts were made to ensure patients were scanned at a standardised time on the day after a dialysis session the natural variation in volume status is this population is likely to confound strain measures and may limit the use of this technique in this population. Although, there are no empirical data to support or refute this hypothesis. In addition, whilst all our findings are biologically plausible, the cross-sectional design means they cannot imply causality or temporal sequence. The SONG-HD initiative emphasised how patients on haemodialysis prioritise interventions that improve lifestyle outcomes, such as mobility and energy, although these are rarely reported as primary outcomes in HD trials [10]. By focussing on physical functioning, we aimed to investigate an outcome that could perceptibly affect a person’s quality of life, in line with the recommendations from this initiative.

## Conclusion

Our study demonstrated that in patients on haemodialysis, native T1 and NT pro-BNP are independent CV determinants of performance in the ISWT and STS60. In addition, LV ejection fraction also significantly determines ISWT performance. Our data enables better understanding of the mechanisms behind the observed CV changes in exercise interventions and could strengthen future work aiming to target core lifestyle outcomes. Furthermore, whilst it is well known that improving physical function improves CV risk, we have been able to pinpoint independent CV biomarkers in that relationship, which may be amenable to improvement. The resounding impact of diabetes on physical functioning in these patients underscores the consequences of metabolic ageing and physical deconditioning. Improving strategies for prevention and management of diabetes may ameliorate the ‘deconditioning spiral’ in these patients. This not only has potential to improve the multimorbidity burden associated with diabetes in ESKD, but importantly, to target lifestyle and physical functioning in these patients.

## Electronic Supplementary Material

Below is the link to the electronic supplementary material.

(DOCX 61 kb)

## Data Availability

The data underlying this article will be shared on reasonable request to the corresponding author.

## Abbreviations

CV: Cardiovascular
CVD: Cardiovascular disease
CMR: Cardiovascular magnetic resonance
CKD: Chronic kidney disease
ESKD: End-stage kidney disease
hsTnI: High-sensitivity troponin I
ISWT: Incremental shuttle walk test
LV: Left ventricle
LV ejection fraction: Left ventricular ejection fraction
LVH: Left ventricular hypertrophy
LVMi: Left ventricular mass index
MOLLI: Modified look-locker inversion recover
NT-proBNP: N-Terminal Pro-BNP
PMV: Pulse wave velocity
STS60: Sit-to-stand 60
