# Supplementary material for: The cardiovascular determinants of physical function in patients with end-stage kidney disease on haemodialysis
Source: Int J Cardiovasc Imaging. 2020 Nov 30;37(4):1405–14. doi: 10.1007/s10554-020-02112-z (PMC8026413; doi:10.1007/s10554-020-02112-z)
Supplement: Supplementary file 1 — (DOCX 61 kb) [file 10554_2020_2112_MOESM1_ESM.docx]

## Supplementary Data

### Appendix 1

#### Average field test performance by subgroup

Table A- 1: Subgroup analysis of average ISWT performance

| **Subgroup** | **n=114** | **Average ISWT performance (m)** | **P-value** |
| --- | --- | --- | --- |
| Male, n (%) | 82 (72) | 278 ± 20 |  |
| Female, n (%) | 32 (28) | 200 (140, 340) | 0.29 |
| White, n (%) | 51 (45) | 270 ± 250 |  |
| BAME, n (%) | 63 (55) | 210 (140, 350) | 0.61 |
| Hypertensive, n (%) | 72 (63) | 275 (152, 370) |  |
| Not hypertensive, n (%) | 42 (37) | 165 (78, 352) | 0.05 |
| Ischaemic heart disease, n (%) | 15 (13) | 229 ± 160 |  |
| No ischaemic heart disease, n (%) | 99 (87) | 240 (140, 370) | 0.34 |
| Diabetic, n (%) | 72 (63) | 172 ±115 |  |
| Not diabetic, n (%) | 41 (36) | 320 (170, 440) | **0.01** |

Normally distributed data presented as mean ± SD, non-normally distributed data presented as median (25th, 75th percentile).

ISWT, incremental shuttle walk test; BAME, Black, Asian and minority ethnic

Table A- 2: Subgroup analysis of average STS60 performance

| **Subgroup** | **n=117** | **Average STS60 performance (reps)** | **P-value** |
| --- | --- | --- | --- |
| Male, n (%) | 82 (70) | 16 (7, 25) |  |
| Female, n (%) | 35 (30) | 15 ± 11 | 0.69 |
| White, n (%) | 54 (46) | 19 (3, 24) |  |
| BAME, n (%) | 63 (54) | 14 (8, 24) | 0.87 |
| Hypertensive, n (%) | 77 (66) | 17 ± 11 |  |
| Not hypertensive, n (%) | 40 (34) | 15 ± 12 | 0.28 |
| Ischaemic heart disease, n (%) | 16 (14) | 13 (5, 23) |  |
| No ischaemic heart disease, n (%) | 101 (86) | 16 ± 11 | 0.45 |
| Diabetic, n (%) | 74 (63) | 12 (0, 19) |  |
| Not diabetic, n (%) | 42 (36) | 19 ± 12 | **0.01** |

Normally distributed data presented as mean ± SD, non-normally distributed data presented as median (25th, 75th percentile).

STS60, sit to stand 60; BAME, Black, Asian and minority ethnic

#### Average values of CV biomarkers by subgroup

Table A- 3: Average left ventricular mass index (LVMi) by subgroup

| **Subgroup** | **n= 130** | **LVMi (g/m^2^)** | **P-value** |
| --- | --- | --- | --- |
| Male, n (%) | 95 (73) | 63 (52, 81) |  |
| Female, n (%) | 35 (27) | 55 ± 17 | **<0.01** |
| White, n (%) | 58 (45) | 63 ± 19 |  |
| BAME, n (%) | 72 (55) | 61 (50, 78) | 0.56 |
| Hypertensive, n (%) | 87 (67) | 63 (50, 81) |  |
| Not hypertensive, n (%) | 43 (33) | 57 (49, 69) | 0.10 |
| Ischaemic heart disease, n (%) | 17 (13) | 63 ± 21 |  |
| No ischaemic heart disease, n (%) | 113 (87) | 62 (50, 77) | 0.53 |
| Diabetic, n (%) | 49 (38) | 64 ± 16 |  |
| Not diabetic, n (%) | 80 (62) | 58 (49, 76) | 0.47 |

Normally distributed data presented as mean ± SD, non-normally distributed data presented as median (25th, 75th percentile).

LVMi, Left ventricular mass index; BAME, Black, Asian and minority ethnic

Table A- 4: Average left ventricular ejection fraction by subgroup

| **Subgroup** | **n= 130** | **LV ejection fraction (%)** | **P-value** |
| --- | --- | --- | --- |
| Male, n (%) | 95 (73) | 53 (46, 58) |  |
| Female, n (%) | 35 (27) | 61 (55, 64) | **<0.01** |
| White, n (%) | 58 (45) | 54 ± 10 |  |
| BAME, n (%) | 72 (55) | 56 (49, 60) | 0.55 |
| Hypertensive, n (%) | 87 (67) | 55 (48, 61) |  |
| Not hypertensive, n (%) | 43 (33) | 54 ± 8 | 0.93 |
| Ischaemic heart disease, n (%) | 17 (13) | 55 (48, 59) |  |
| No ischaemic heart disease, n (%) | 113 (87) | 56 (47, 61) | 0.53 |
| Diabetic, n (%) | 49 (38) | 52 ± 9 |  |
| Not diabetic, n (%) | 80 (62) | 56 (50, 61) | 0.22 |

Normally distributed data presented as mean ± SD, non-normally distributed data presented as median (25th, 75th percentile).

LV, left ventricular; BAME, Black, Asian and minority ethnic

Table A- 5: Average left ventricular mass/volume ratio by subgroup

| **Subgroup** | **n= 130** | **LV mass/LV end-diastolic volume (g/mL)** | **P-value** |
| --- | --- | --- | --- |
| Male, n (%) | 95 (73) | 0.75 ± 0.17 |  |
| Female, n (%) | 35 (27) | 0.69 ± 0.13 | 0.08 |
| White, n (%) | 58 (45) | 0.74 ± 0.18 |  |
| BAME, n (%) | 72 (55) | 0.72 ± 0.14 | 0.51 |
| Hypertensive, n (%) | 87 (67) | 0.73 (0.62, 0.83) |  |
| Not hypertensive, n (%) | 43 (33) | 0.72 ± 0.17 | 0.68 |
| Ischaemic heart disease, n (%) | 17 (13) | 0.69 ± 0.11 |  |
| No ischaemic heart disease, n (%) | 113 (87) | 0.74 ± 0.16 | 0.27 |
| Diabetic, n (%) | 49 (38) | 0.76 ± 0.17 |  |
| Not diabetic, n (%) | 80 (62) | 0.71 ± 0.15 | 0.12 |

Normally distributed data presented as mean ± SD, non-normally distributed data presented as median (25th, 75th percentile).

LV, left ventricular; BAME, Black, Asian and minority ethnic

Table A- 6: Average global native T1 by subgroup

| **Subgroup** | **n= 124** | **Global Native T1 (ms)** | **P-value** |
| --- | --- | --- | --- |
| Male, n (%) | 91 (73) | 1276 ± 42 |  |
| Female, n (%) | 33 (27) | 1267 ± 37 | 0.09 |
| White, n (%) | 54 (44) | 1278 ± 45.8 |  |
| BAME, n (%) | 70 (56) | 1272 (1246, 1289) | 0.47 |
| Hypertensive, n (%) | 84 (68) | 1274 ± 42 |  |
| Not hypertensive, n (%) | 40 (32) | 1274 ± 40 | 0.98 |
| Ischaemic heart disease, n (%) | 17 (14) | 1263 ± 30 |  |
| No ischaemic heart disease, n (%) | 107 (86) | 1275 ± 42 | 0.24 |
| Diabetic, n (%) | 48 (39) | 1269 (1246, 1301) |  |
| Not diabetic, n (%) | 75 (60) | 1271 ± 40 | 0.68 |

Normally distributed data presented as mean ± SD, non-normally distributed data presented as median (25th, 75th percentile).

BAME, Black, Asian and minority ethnic

Table A- 7: Average global longitudinal strain by subgroup

| **Subgroup** | **n= 130** | **Global longitudinal strain (%)** | **P-value** |
| --- | --- | --- | --- |
| Male, n (%) | 95 (73) | -13 (-15, -10) |  |
| Female, n (%) | 35 (27) | -15 ± 2 | **<0.01** |
| White, n (%) | 58 (45) | -13 ± 3 |  |
| BAME, n (%) | 72 (55) | -14 (-16, -11) | 0.81 |
| Hypertensive, n (%) | 87 (67) | -14 (-15, -11) |  |
| Not hypertensive, n (%) | 43 (33) | -13 ± 3 | 0.99 |
| Ischaemic heart disease, n (%) | 17 (13) | -14 (-15, -10) |  |
| No ischaemic heart disease, n (%) | 113 (87) | -13 ± 3 | 0.32 |
| Diabetic, n (%) | 49 (38) | -13 ± 3 |  |
| Not diabetic, n (%) | 80 (62) | -14 (-16, -11) | 0.12 |

Normally distributed data presented as mean ± SD, non-normally distributed data presented as median (25th, 75th percentile).

BAME, Black, Asian and minority ethnic

Table A- 8: Average pulse wave velocity (PWV) by subgroup

| **Subgroup** | **n= 117** | **PWV (m/s)** | **P-value** |
| --- | --- | --- | --- |
| Male, n (%) | 86 (74) | 8 (6, 12) |  |
| Female, n (%) | 31 (26) | 7 (5, 10) | 0.26 |
| White, n (%) | 52 (44) | 8 (6, 11) |  |
| BAME, n (%) | 65 (55) | 8 (6, 11) | 0.47 |
| Hypertensive, n (%) | 79 (68) | 8 (6, 12) |  |
| Not hypertensive, n (%) | 38 (32) | 8 (6, 10) | 0.64 |
| Ischaemic heart disease, n (%) | 15 (13) | 8 ± 3 |  |
| No ischaemic heart disease, n (%) | 102 (87) | 8 (6, 11) | 0.44 |
| Diabetic, n (%) | 45 (38) | 9 (7, 12) |  |
| Not diabetic, n (%) | 71 (61) | 7 (6, 10) | **0.04** |

Normally distributed data presented as mean ± SD, non-normally distributed data presented as median (25th, 75th percentile).

PWV, pulse wave velocity; BAME, Black, Asian and minority ethnic

Table A- 9: Average NT pro-BNP by subgroup

Normally distributed data presented as mean ± SD, non-normally distributed data presented as median (25th, 75th percentile).

BAME, Black, Asian and minority ethnic

| **Subgroup** | **n= 120** | **NT pro-BNP (pg/ml)** | **P-value** |
| --- | --- | --- | --- |
| Male, n (%) | 86 (72) | 8 (7, 9) |  |
| Female, n (%) | 34 (28) | 8 ± 2 | 0.96 |
| White, n (%) | 56 (47) | 8 (7, 9) |  |
| BAME, n (%) | 64 (53) | 8 ± 2 | 0.38 |
| Hypertensive, n (%) | 81 (68) | 8 ± 2 |  |
| Not hypertensive, n (%) | 39 (32) | 8 ± 1 | 0.08 |
| Ischaemic heart disease, n (%) | 16 (13) | 8 ± 2 |  |
| No ischaemic heart disease, n (%) | 104 (87) | 8 (7, 9) | 0.93 |
| Diabetic, n (%) | 45 (38) | 8 ± 1 |  |
| Not diabetic, n (%) | 74 (62) | 8 (7, 9) | 0.44 |

Table A- 10: Average high-sensitivity Troponin I by subgroup

Normally distributed data presented as mean ± SD, non-normally distributed data presented as median (25th, 75th percentile).

BAME, Black, Asian and minority ethnic

| **Subgroup** | **n= 124** | **Troponin (ng/L)** | **P-value** |
| --- | --- | --- | --- |
| Male, n (%) | 90 (73) | 11 (7, 17) |  |
| Female, n (%) | 34 (27) | 7 (5, 14) | **0.04** |
| White, n (%) | 57 (46) | 12 (7, 18) |  |
| BAME, n (%) | 67 (54) | 9 (5, 14) | 0.20 |
| Hypertensive, n (%) | 82 (66) | 11 (5, 17) |  |
| Not hypertensive, n (%) | 42 (34) | 10 (6, 15) | 0.56 |
| Ischaemic heart disease, n (%) | 16 (13) | 11 (7, 17) |  |
| No ischaemic heart disease, n (%) | 108 (87) | 10 (6, 17) | 0.64 |
| Diabetic, n (%) | 47 (38) | 12 (8, 24) |  |
| Not diabetic, n (%) | 76 (61) | 8 (5, 15) | **0.01** |

### Appendix 2

*Table A- 11: Spearman correlations between LVEF, NT pro-BNP, global native T1 and LVMi*

|  | LVEF (%) | NT pro-BNP (pg/ml) | Global Native T1 (ms) | LVMi (g/m^2^) |
| --- | --- | --- | --- | --- |
| LVEF (%) | 1.00 |  |  |  |
| NT pro-BNP (pg/ml) | -0.27* | 1.00 |  |  |
| Global Native T1 (ms) | -0.42* | 0.56* | 1.00 |  |
| LVMi (g/m^2^) | -0.37* | 0.49* | 0.24* | 1.00 |

*p<0.01 (2-tailed)

LVEF, Left ventricular ejection fraction; LVMi, Left ventricular mass index

### Appendix 3

Table A- 12: Full multivariate linear regression models between tests of physical performance and biomarkers of cardiovascular health, adjusted for age, gender, body mass index, diabetes, systolic blood pressure and ethnicity.

|  | B (SE) | β | p-value |
| --- | --- | --- | --- |
| Dependent variable: ISWT, R^2^= 0.38 | | | |
| Troponin I (ng/L)^a^ | -16.80 (12.0) | -0.12 | 0.17 |
| Age | -4.79 (0.9) | -0.43 | **<0.01** |
| Gender | -67.26 (29.2) | -0.19 | **0.02** |
| BMI | -0.73 (2.3) | -0.03 | 0.75 |
| Diabetes | -74.29 (29.5) | -0.22 | **0.01** |
| Systolic BP (mmHg) | 0.97 (0.61) | 0.13 | 0.11 |
| Ethnicity | -13.00 (26.2) | -0.04 | 0.62 |
| Dependent variable: STS60, R^2^= 0.33 | | | |
| Troponin I (ng/L) ^a^ | -0.23 (0.8) | -0.03 | 0.78 |
| Age | -0.34 (0.1) | -0.46 | **<0.01** |
| Gender | -1.84 (1.9) | -0.08 | 0.34 |
| BMI | -0.11 (0.2) | -0.06 | 0.47 |
| Diabetes | -4.42 (2.0) | -0.20 | **0.03** |
| Systolic BP (mmHg) | 0.02 (0.04) | 0.05 | 0.56 |
| Ethnicity | -0.63 (1.8) | -0.03 | 0.72 |
| Dependent variable: ISWT, R^2^=0.39 | | | |
| NT pro-BNP (pg/ml) ^a^ | -20.33 (8.9) | -0.19 | **0.02** |
| Age | -4.84 (0.9) | -0.44 | **<0.01** |
| Gender | -58.20 (29.1) | -0.16 | **0.04** |
| BMI | -1.89 (2.6) | -0.06 | 0.47 |
| Diabetes | -78.73 (29.2) | -0.23 | **<0.01** |
| Systolic BP (mmHg) | 0.95 (0.6) | 0.13 | 0.12 |
| Ethnicity | -4.12 (26.4) | -0.01 | 0.88 |
| Dependent variable: STS60, R^2^= 0.35 | | | |
| NT pro-BNP (pg/ml) ^a^ | -1.34 (0.6) | -0.19 | **0.03** |
| Age | -0.32 (0.1) | -0.44 | **<0.01** |
| Gender | -1.38 (1.9) | -0.06 | 0.48 |
| BMI | -0.27 (0.2) | -0.14 | 0.12 |
| Diabetes | -4.47 (2.0) | -0.20 | **0.03** |
| Systolic BP (mmHg) | 0.02 (0.04) | 0.05 | 0.58 |
| Ethnicity | -0.35 (1.8) | -0.02 | 0.84 |
| Dependent variable: ISWT, R^2^= 0.39 | | | |
| LVMi (g/m^2^) | -0.31 (0.7) | -0.04 | 0.67 |
| Age | -5.39 (0.9) | -0.46 | **<0.01** |
| Gender | -74.77 (31.1) | -0.20 | **0.02** |
| BMI | -1.25 (2.3) | -0.04 | 0.59 |
| Diabetes | -86.39 (29.8) | -0.24 | **<0.01** |
| Systolic BP (mmHg) | 0.90 (0.6) | 0.12 | 0.15 |
| Ethnicity | -12.52 (26.5) | -0.04 | 0.64 |
| Dependent variable: STS60, R^2^=0.34 | | | |
| LVMi (g/m^2^) | -0.03 (0.05) | -0.06 | 0.52 |
| Age | -0.35 (0.1) | -0.46 | **<0.01** |
| Gender | -2.20 (2.1) | -0.09 | 0.30 |
| BMI | -0.15 (0.2) | -0.08 | 0.35 |
| Diabetes | -5.08 (2.1) | -0.21 | **0.02** |
| Systolic BP (mmHg) | 0.03 (0.04) | 0.06 | 0.50 |
| Ethnicity | 0.01 (1.8) | 0.00 | 0.99 |
| Dependent variable: ISWT, R^2^=0.43 | | | |
| LV ejection fraction (%) | 3.74 (1.4) | 0.21 | **0.01** |
| Age | -5.14 (0.9) | -0.46 | **<0.01** |
| Gender | -92.54 (30.1) | -0.24 | **<0.01** |
| BMI | -1.18 (2.3) | -0.04 | 0.60 |
| Diabetes | -82.24 (28.8) | -0.23 | **<0.01** |
| Systolic BP (mmHg) | 1.03 (0.6) | 0.13 | 0.09 |
| Ethnicity | -9.69 (25.7) | -0.03 | 0.71 |
| Dependent variable: STS60, R^2^=0.35 | | | |
| LV ejection fraction (%) | 0.14 (0.1) | 0.12 | 0.15 |
| Age | -0.33 (0.1) | -0.43 | **<0.01** |
| Gender | -2.74 (2.1) | -0.11 | 0.20 |
| BMI | -0.15 (0.2) | -0.08 | 0.35 |
| Diabetes | -5.13(2.1) | -0.22 | **0.01** |
| Systolic BP (mmHg) | 0.03 (0.04) | 0.06 | 0.45 |
| Ethnicity | 0.17 (1.8) | 0.01 | 0.93 |
| Dependent variable: ISWT, R^2^= 0.39 | | | |
| PWV (m/s) | -1.10 (3.2) | -0.03 | 0.74 |
| Age | -4.89 (1.1) | -0.44 | **<0.01** |
| Gender | -79.89 (32.0) | -0.21 | **0.01** |
| BMI | 0.14 (2.6) | 0.04 | 0.99 |
| Diabetes | -95.26 (32.1) | -0.27 | **<0.01** |
| Systolic BP (mmHg) | 0.65 (0.7) | 0.08 | 0.32 |
| Ethnicity | -10.57 (28.4) | -0.03 | 0.71 |
| Dependent variable: STS60, R^2^= 0.33 | | | |
| PWV (m/s) | 0.11 (0.2) | 0.05 | 0.64 |
| Age | -0.33 (0.1) | -0.44 | **<0.01** |
| Gender | -2.47 (2.1) | -0.10 | 0.25 |
| BMI | -0.07 (0.2) | -0.03 | 0.71 |
| Diabetes | -6.03 (2.2) | -0.26 | **<0.01** |
| Systolic BP (mmHg) | 0.02 (0.04) | 0.05 | 0.60 |
| Ethnicity | 0.44 (1.9) | 0.02 | 0.64 |
| Dependent variable: ISWT, R^2^=0.39 | | | |
| LV mass:volume (g/mL) | -46.93 (82.9) | -0.05 | 0.57 |
| Age | -5.29 (0.9) | -0.47 | **<0.01** |
| Gender | -71.78 (30.18) | -0.19 | **0.02** |
| BMI | -1.05 (2.3) | -0.04 | 0.65 |
| Diabetes | -86.26 (29.8) | -0.24 | **<0.01** |
| Systolic BP (mmHg) | 0.82(0.6) | 0.11 | 0.19 |
| Ethnicity | -13.69 (26.6) | -0.04 | 0.61 |
| Dependent variable: STS60, R^2^=0.33 | | | |
| LV mass:volume (g/mL) | -0.45 (5.7) | -0.01 | 0.94 |
| Age | -0.34 (0.1) | -0.45 | **<0.01** |
| Gender | -1.78 (2.0) | -0.07 | 0.38 |
| BMI | -0.14 (0.2) | -0.07 | 0.39 |
| Diabetes | -5.21 (2.1) | -0.22 | **0.01** |
| Systolic BP (mmHg) | 0.03 (0.04) | 0.05 | 0.54 |
| Ethnicity | 0.03 (1.8) | 0.001 | 0.99 |
| Dependent variable: ISWT, R^2^= 0.48 | | | |
| Global Native T1 (ms) | -1.29 (0.3) | -0.29 | **<0.01** |
| Age | -4.95 (0.86) | -0.45 | **<0.01** |
| Gender | -73.24 (28.4) | -0.19 | **0.01** |
| BMI | -0.47 (2.2) | -0.02 | 0.83 |
| Diabetes | -89.32 (27.8) | -0.25 | **<0.01** |
| Systolic BP (mmHg) | 1.04 (0.6) | 0.13 | 0.08 |
| Ethnicity | -29.31 (25.3) | -0.09 | 0.25 |
| Dependent variable: STS60, R^2^=0.36 | | | |
| Global Native T1 (ms) | -0.06 (0.02) | -0.19 | **0.02** |
| Age | -0.31 (0.1) | -0.42 | **<0.01** |
| Gender | -2.10 (2.0) | -0.08 | 0.31 |
| BMI | -0.13 (0.2) | -0.07 | 0.41 |
| Diabetes | -5.37 (2.1) | -0.23 | **0.01** |
| Systolic BP (mmHg) | 0.03 (0.04) | 0.06 | 0.47 |
| Ethnicity | -0.71 (1.9) | -0.03 | 0.70 |
| Dependent variable: ISWT, R^2^=0.41 | | | |
| Global longitudinal strain (%) | -7.97 (4.3) | -0.15 | 0.07 |
| Age | -5.10 (0.9) | -0.45 | **<0.01** |
| Gender | -83.76 (30.4) | -0.22 | **<0.01** |
| BMI | -0.84 (2.3) | -0.03 | 0.71 |
| Diabetes | -84.14 (29.3) | -0.24 | **<0.01** |
| Systolic BP (mmHg) | 1.04 (0.6) | 0.13) | 0.10 |
| Ethnicity | -10.10 (26.1) | -0.03 | 0.70 |
| Dependent variable: STS60, R^2^=0.34 | | | |
| Global longitudinal strain (%) | -0.27 (0.3) | -0.08 | 0.35 |
| Age | -0.33 (0.1) | -0.44 | **<0.01** |
| Gender | -2.32 (2.1) | -0.09 | 0.26 |
| BMI | -0.13 (0.16) | -0.07 | 0.38 |
| Diabetes | -5.15 (2.1) | -0.22 | **<0.01** |
| Systolic BP (mmHg) | 0.03 (0.04) | 0.06 | 0.45 |
| Ethnicity | 0.11 (1.8) | 0.01 | 0.95 |

^a^ Log transformed data. B=unstandardized beta coefficient; SE= standard error of the mean; β= standardized beta coefficient;

The reference category for gender is ‘male’, for history of diabetes is ‘no’ and for ethnicity is ‘white’.

Abbreviations: BMI, body mass index; ISWT, incremental shuttle walk test; STS60, sit-to-stand 60; LVMi, left ventricular mass index; LV, left ventricle; PWV, pulse wave velocity; BP, blood pressure.
